# Development of butanol-tolerant *Bacillus subtilis *strain GRSW2-B1 as a potential bioproduction host

**DOI:** 10.1186/2191-0855-1-10

**Published:** 2011-05-30

**Authors:** Naoya Kataoka, Takahisa Tajima, Junichi Kato, Wanitcha Rachadech, Alisa S Vangnai

**Affiliations:** 1 Department of Molecular Biotechnology, Graduate School of Advanced Sciences of Matter, Hiroshima University, Hiroshima 739-8530, Japan; 2 Department of Biochemistry, Faculty of Science, Chulalongkorn University, Bangkok 10330, Thailand; 3 National Center of Excellence for Environmental and Hazardous Waste Management (NCE-EHWM), Chulalongkorn University, Bangkok 10330 Thailand

**Keywords:** Organic-solvent tolerant bacteria, Butanol-tolerant bacteria, Heterologous gene-expression host

## Abstract

As alternative microbial hosts for butanol production with organic-solvent tolerant trait are in high demands, a butanol-tolerant bacterium, *Bacillus subtilis *GRSW2-B1, was thus isolated. Its tolerance covered a range of organic solvents at high concentration (5%v/v), with remarkable tolerance in particular to butanol and alcohol groups. It was susceptible for butanol acclimatization, which resulted in significant tolerance improvement. It has versatility for application in a variety of fermentation process because it has superior tolerance when cells were exposed to butanol either as high-density, late-exponential grown cells (up to 5%v/v) or under growing conditions (up to 2.25%v/v). Genetic transformation procedure was optimized, yielding the highest efficiency at 5.17 × 10^3 ^colony forming unit (μg DNA)^-1^. Gene expression could be effectively driven by several promoters with different levels, where as the highest expression was observed with a xylose promoter. The constructed vector was stably maintained in the transformants, in the presence or absence of butanol stress. Adverse effect of efflux-mediated tetracycline resistance determinant (TetL) to bacterial organic-solvent tolerance property was unexpectedly observed and thus discussed. Overall results indicate that *B. subtilis *GRSW2-B1 has potential to be engineered and further established as a genetic host for bioproduction of butanol.

## Introduction

*n*-Butanol (hereafter referred to as butanol) is an important industrial chemical, widely used as a solvent, a stabilizer and feedstock for the production of polymers and plastics. Recently, butanol has been considered as a potential advanced biofuel with several advantages over ethanol because it contains higher energy density, lower vapor pressure, less corrosive and less water solubility (Connor and Liao 2009). Due to a limited supply of petroleum oil, microbial production of butanol has gained more attentions in present years. However, major roadblocks of the current butanol fermentation are low yield, low productivity and, most importantly, low titer due to the toxicity of butanol to its producing strains (Liu and Qureshi 2009). Generally, butanol inhibits microbial growth, including growth of current butanol-producing *Clostridium *strains, when the concentration reaches 2%v/v (*ca*. 16 g L^-1^). Butanol sensitivity and complex regulatory pathways of *Clostridium *strains are the key restrictions to the progress of butanol fermentation in the native host. Therefore, an alternative approach for butanol production is to find and construct butanol biosynthesis pathway in a heterologous host, of which one of the crucial considerable characteristics is butanol tolerance (Liu and Qureshi 2009). So far, alternative hosts being engineered for butanol production are well-characterized, genetically-amenable microorganisms, such as *Escherichia coli *([Bibr B2][Bibr B13]; Nielsen et al. 2009), *Saccharomyces cerevisiae *(Steen et al. 2008), *Clostridium ljungdahlii *(Kopke et al. 2010) and organic-solvent tolerant bacteria (OSTB), such as *Pseudomonas putida *S12 and *Bacillus subtilis *KS438 (Nielsen et al. 2009). They were capable of producing butanol, although at relatively low yield, but the critical remaining problem was that they still severely suffer from butanol toxicity as their viability was significantly decreased at 0.75, 1.0, 1.25, 2.0%v/v butanol for *P. putida*, *E. coli*, *B. subtilis*, (Nielsen et al. 2009), *S. cerevisiae *(Liu and Qureshi 2009) and *Clostridia *(Ezeji et al. 2010), respectively. Therefore, it is obviously shown that butanol tolerance is one of the important traits, if not the most, in selecting host and thus several studies have been conducted to search for butanol-tolerant microorganisms (Fischer et al. 2008;Knoshaug and Zhang 2009). Nevertheless, to be suitable as a potential genetic engineered host for bioproduction of chemicals, other fundamental, but requisite, knowledge of the host regarding genetic competency, gene expression strength, etc. should be proven feasible.

In this study, *Bacillus subtilis *strain GRSW2-B1 was isolated as a butanol-tolerant bacterium. It exhibited tolerance to butanol and other organic solvents (referred to as solvent hereafter) at relatively high concentrations. To further develop this strain to be a genetic host for bioproduction of solvent-type chemicals, including butanol, the genetic manipulation and genetic characteristics were investigated and optimized. In addition, this study is the first to report the negative influence of efflux-mediated tetracycline resistance determinant (TetL) on bacterial organic-solvent tolerance.

## Materials and Methods

### Chemicals and cultivation medium

Solvents and culture medium components were from Nacalai Tesque Inc (Kyoto, Japan). All reagents used were analytical grade. Bacterial cultivation medium was either Luria-Bertani (LB) medium or minimal salt basal medium (MSB) (Kongpol et al. 2008). Chemical reagents and enzymes (e.g. KOD plus, Ligation-High, etc.) for molecular biology protocols were from Toyobo, Inc (Japan) unless stated otherwise.

### Isolation, identification and characterization of butanol-tolerant bacteria

Bacteria were screened from seawater samples from several areas in Thailand. Seawater samples were mixed with Luria-Bertani (LB) medium and incubated at room temperature (~33°C) for 8 h. Butanol was then provided at 0.1%v/v, incubated overnight before the bacterial culture was diluted and plated onto LB medium agar to obtain single colonies. The isolates with different colony morphologies were examined for their tolerance to butanol at 1%v/v, and then selected for further investigations. The selected bacterial isolate was identified by morphology observation and 16S rRNA sequence analysis according to (Kongpol et al (2008)). The partial sequence of 16S rRNA gene was analyzed using BLASTN program and submitted to the GenBank nucleotide sequence database (NCBI) [GenBank:HQ912916]. The strain was deposited to Thailand culture collection (BIOTEC, Thailand) with the biological material number BCC45739. Growth characteristic of the selected isolate was determined under various conditions including carbon source (glucose (4 g L^-1^), xylose (4 g L^-1^), butanol (0.1 and 0.5%v/v) in MSB medium, temperature (28, 37, 45°C) and salinity (0.5-14% NaCl).

### Organic-solvent tolerance

Solvent tolerance characteristic was conducted by two procedures. First, cells were grown in LB medium at 37°C, 120 rpm to late-exponential phase. Then, solvent was directly added to 5%v/v, exposed to a high-density cell for 6 h and cell viability was determined as colony-forming unit per milliliter (CFU ml^-1^). Second, to test tolerance of growing culture, butanol at various concentrations (1.5-2.25% v/v) was added simultaneously with the bacterial inoculum in LB medium. Then, cell growth determined as cell optical density at 600 nm (OD_600_) was used as a parameter for cell viability and tolerance.

### Cell acclimatization to butanol

The selected isolate was grown in LB medium supplemented with butanol (1.5%v/v) at 37°C for 12 h (representing one acclimatization cycle) used as cell inoculum (1.5%v/v) for subsequent batch. Cells, which were acclimatized for 30 cycles, were then tested for butanol tolerance (up to 2.25%v/v).

### Preparation of electro-competent cells and electroporation conditions

The selected isolate was grown in LB medium at 37°C to three different growth stages monitored by OD_600 _(i.e. early-exponential phase, 0.3; mid-exponential phase, 0.6; late-exponential phase, 0.9). Cells were chilled on ice for 10 min before harvesting, washed four times with ice-cold electroporation media (sterile distilled water, glycerol solution [10% v/v], HS buffer [250 mM sucrose, 1 mM HEPES, pH 7.0] or HSMG buffer [HS buffer with 1 mM MgCl_2 _and 10% glycerol, pH 7.0] (Turgeon et al. 2006), and concentrated 150-fold.

Then, competent cells (0.1 ml) were mixed with pHY300PLK plasmid DNA (Takara Bio Inc., Japan) at various concentrations of (50, 100, 200, 500, 100, 1000 ng μl^-1^) and kept on ice for 20 min. Electroporation was performed in 2-mm gapped BTX electroporation cuvette Plus™ at 25 μF, 200 Ω with various pulse strengths (8, 9, 10, 10.5, 11, 12 kV cm^-1^) using Electro Cell Manipulator, model ECM 630 (BTX Molecular Delivery Systems, Harvard Apparatus Inc., CA, USA). Pulsed cells were immediately diluted with 1 ml of either Tryptic Soy Broth (TSB) medium or TSB supplemented with 5 mM MgCl_2_, 5 mM MgSO_4_, and 250 mM sucrose (TSB-plus medium) and incubated during recovery period with shaking (120 rpm) for 2 or 3 h before spreading on LB medium agar plate including tetracycline (10 μg ml^-1^) or kanamycin (5 μg ml^-1^) as indicated.

### Construction of plasmids

To assess the promoter activities of several promoters in *B. subtilis *GRSW2-B1, pHY300PLK derivatives containing the promoter::*lacZ *transcriptional fusion genes were constructed (Table 1). Promoter regions were amplified by PCR. Primers and template DNA used for PCR amplification are shown in Table 2. Amplified products were digested with *Sph*I and *Hin*dIII, and cloned between *Sph*I and *Hin*dIII sites of pQF50, a Gram-negative promoterless *lacZ *transcriptional fusion vector (Farinha and Kropinski 1990), to construct promoter::*lacZ *transcriptional fusion genes. A PCR product for the promoter P*_xylA _*was digested with *Sph*I and *Xba*I and cloned between *Sph*I and *Xba*I sites of pQF50. The promoter::*lacZ *transcriptional fusion genes were then amplified by PCR with Z-F/Z-R as primers and pQF50 derivatives as templates. Amplified products were digested with *Bgl*II and cloned between *Sma*I and *Bgl*II of pHY300PLK to construct pHY300PLK derivatives containing the promoter::*lac*Z transcriptional fusion genes, i.e. pHZT-P43, pHZT-P2N, pHZT-P2L, pHZT-PT, pHZT-PS, and pHZT-PX. The promoterless *lacZ *was amplified from pQF50 by PCR with Z-F/Z-R as primers and the resulting product was digested with *Bgl*II, and cloned between *Sma*I and *Bgl*II sites of pHY300PLK to construct control plasmid pHZT. Plasmid pHZK-PX was a pHZT-PX derivative, in which tetracycline resistant gene (*tetL*) was substituted with kanamycin resistant gene (*kan*) from pDG148. To amplify pHZT-PX DNA region without the *tetL *gene, PCR was conducted using TZ-F/TZ-R as primers and pHZT-PX as a template, and the *kan *gene was amplified from pDG148 using K-F/K-R primers. The resulting PCR products were joined using In-Fusion^® ^Advantage PCR cloning kit (Clontech, Japan).

**Table 1:** Bacterial strains and plasmids used in this study

Bacterial strain or plasmid	Relevant characteristic(s)	Source or reference
*B. subtilis *GRSW2-B1	Butanol-tolerant bacterium	This study
*B. subtilis *168	A type-strain *Bacillus subtilis*. Source of promoter sequences: P_43_, P_2L_	Laboratory stock
*E. coli *DH5α	*hsdR17 recA endA1 lacZΔM15*. For plasmid construction and propagation purpose	Invitrogen, USA
Plasmids		
pHY300PLK	A shuttle vector for *E. coli *and *B. subtilis*, carrying *bla *(Ap^r^) and *tetL *(Tc^r^). Source of tetracycline promoter (P_Tet_)	(Ishiwa and Shibahara 1985)
pQF50	A broad-host range vector. Source of *trpA *terminators, a multiple cloning site (MCS) and *lacZ*	(Farinha and Kropinski 1990)
pUC4K	A vector carrying Ap^r^, Km^r^. Source of kanamycin promoter (P_Km_)	Laboratory stock
pNCMO2	A vector carrying strong promoter P2 for *Brevibacillus*. Source of P2 promoter (P_2N_)	Takara Bio Inc., Japan
pDG148	A shuttle vector for *E. coli *and *B. subtilis*, carrying *bla *(Ap^r^) and *kan *(Km^r^). Source of kanamycin resistant gene cassette and Spac promoter (P_Spac_)	Laboratory stock
pWH1520	An expression vector for *B. megaterium*. Source of xylose promoter (P_xylA_)	Mo Bi Tec, Germany
pHZT	pHY300PLK (Tc^r^) carrying *trpA*, MCS, *lacZ*	This study
pHZK	pHY300PLK, carrying *trpA*, MCS, *lacZ*, and *tetL *(Tc^r^) was replaced with *kan *(Km^r^)	This study
pHZT-P43	pHZT carrying P_43_	This study
pHZT-PK	pHZT carrying P_Km_	This study
pHZT-P2N	pHZT carrying P_2N_	This study
pHZT-P2L	pHZT carrying P_2L_	This study
pHZT-PT	pHZT carrying P_Tet_	This study
pHZT-PS	pHZT carrying P_Spac_	This study
pHZT-PX	pHZT carrying P_xylA_	This study
pHZK-PX	pHZK carrying P_xylA_	This study

**Table 2:** Primers and source of sequence

Region description	Primer	Primer sequence (5' → 3')^a^	Source of sequence or reference
Terminator -LacZ	Z-F	CTCTGATGCCGCATAGTTAA	pQF50, Laboratory stock
	Z-R	**ctag**AGATCT(BglII)CATAATGGATTTCCTTACGC	
P43 promoter	P43-F	**GCAG**GCATGC(SphI)ACTGACAAACATCACCCTCT	*B. subtilis *168 chromosome
	P43-R	**aTgc**AAGCTT(HindIII)TGGTACCGCTATCACTTTAT	
P_Km _promoter	P_Km_-F	**gcag**GCATGC(SphI)GCTATGACCATGATTACGAA	pUC4K, Laboratory stock
	P_Km_-R	**aTgc**AAGCTT(HindIII)TGTATTACTGTTTATGTAAGCAGAC	
P_2N _promoter	P_2N_-F	**GCAG**GCATGC(SphI)TCACTTCGTACATAATGGAC	pNCMO2, Takara Bio Inc, Japan
	P_2N_-R	**ATGC**AAGCTT(HindIII)TTCGCAGGAAAGCCATG	
P_2L _promoter	P_2L_-F	**GCAG**GCATGC(SphI)GATCAGCTTGAAATATGTACATAG	*B. subtilis *168 chromosome
	P_2L_-R	**ATGC**AAGCTT(HindIII)TGATAAATTTATTTATTTAGGATCCGATCT	
P_Tet _promoter	P_Tet_-F	**gcag**GCATGC(SphI)GTTCAACAAACGGGCCATAT	pHY300PLK, Takara Bio Inc, Japan
	P_Tet_-R	**aTgc**AAGCTT(HindIII)AATAATGAGGGCAGACGTAG	
P_spac _promoter	P_spac_-F	**GCAG**GCATGC(SphI)CGCACCCTGAAGAAGATTTA	pDG148, Laboratory stock
	P_spac_-R	**ATGC**AAGCTT(HindIII)AATTGTTATCCGCTCA	
P_xylA _promoter	P_xyl_-F	**gcag**GCATGC(SphI)ATCCACCGAACTAAGTTGGT	pWH1520, Mo Bi Tec, Germany
	P_xyl_-R	**ATcc**TCTAGA(XbaI)TTGATTTAAGTGAACAAGTTTATCCATC	
pHY300PLK *ΔtetL*	TZ-F	ATCGTTAAGGGATCAACTTTGGGAG	pHY300PLK, Takara Bio Inc, Japan
	TZ-R	ATTTCACCCTCCAATAATGAGGGC	
Km^r ^(*kan*)	K-F	ATTGGAGGGTGAAATATGAGAATAGTGAATGGACCAA	pDG148, Laboratory stock
	K-R	TGATCCCTTAACGATTCAAAATGGTATGCGTTTTGAC	
16s rRNA	63-F	CAGGCCTAACACATGCAAGTC	(Marchesi et al. 1998)
	1387-R	GGGCGGWGTGTACAAGGC	

a Additional nucleotides are shown in boldface; Recognition sequences of restriction enzymes are underlined and shown in parenthesis

### Determination of segregational stability of plasmid

Segregational stability of plasmid was evaluated by growing *B. subtilis *GRSW2-B1 harboring pHZK-PX in the LB medium, without kanamycin, in the presence and absence of butanol (1%v/v), for two generations. Aliquots were withdrawn from each generation and plated on LB medium agar and replica plated on LB medium agar containing kanamycin (5 μg ml^-1^). The percentage of segregational stability of the plasmid was calculated from [number of colonies on the plate without antibiotic] divided by [number of colonies on the plate with antibiotic] × 100.

### Assay of β-galactosidase activity

β-Galactosidase activity was quantitatively assayed according to the method previously reported (Rygus and Hillen 1991). Briefly, cells were grown in LB medium containing an appropriate antibiotic to reach OD_600 _of 0.8 and were permeabilized with toluene (2%v/v). If the induction was needed, the inducer was added when OD_600 _was at 0.3. One unit of β-galactosidase activity was calculated according to Miller (Miller 1972).

## Results

### Isolation of butanol-tolerant bacteria

Most Gram negative OSTB have been isolated from soil samples, but a greater biodiversity of OSTB has been described in the marine environment because the relatively high salt concentration may induce multidrug efflux pump activity in bacteria, leading to their higher solvent tolerance (Sardessai and Bhosle 2002). In this study, butanol-tolerant bacteria were screened from seawater samples with butanol enrichment (0.1%v/v). Nine marine bacterial isolates obtained - one being *Exiguobacterium *sp. and the rest belonging to *Bacillus *sp - were further tested for their tolerance to butanol at 1%v/v (data not shown). Four of them (GRSW1-B1, GRSW2-B1, CPSW1-B1 and CPSW2-B1) exhibited relatively good tolerance at 1%v/v, but due to the limitation of genetic transformation feasibility (as described later), isolate GRSW2-B1 was selected for further investigation. Isolate GRSW2-B1 is a Gram-positive, endospore-forming bacterium. The analysis of a partial sequence of 16S rRNA indicates that it is identical to *Bacillus subtilis*. Thus, we refer to this isolate as *B. subtilis *GRSW2-B1 or GRSW2-B1 hereafter.

### Characterization of the selected butanol-tolerant bacterium GRSW2-B1

The fact that the selected butanol-tolerant bacterium is *B. subtilis *is beneficial for the development of an expression host for bioproduction. *B. subtilis *is generally considered as an industrial strain, which is also suitable as a host organism, because it is a non-pathogenic organism that has the secretory capacity to export proteins into the extracellular medium (advantageous for heterologous protein synthesis). In addition, its genome database is available and it is a genetically amenable host organism for which genetic tools are readily available (Fischer et al. 2008). Nevertheless, prior to further development of GRSW2-B1 as a genetic recombinant host, it is essential to gain fundamental knowledge of its growth conditions and, most importantly, its butanol tolerance characteristics.

GRSW2-B1 was able to utilize glucose (4 g L^-1^) and xylose (4 g L^-1^) as carbon sources in MSB medium at 37°C, exhibiting growth rates of 0.052 ± 0.021 h^-1 ^and 0.013 ± 0.006 h^-1^, respectively. It could not utilize butanol as a sole carbon source when butanol was supplemented at non-lethal concentrations (0.1%v/v and 0.5%v/v) in MSB medium. It could grow at a temperature ranging from 28-45°C and had an approximately similar maximum growth rate of 0.497 ± 0.007 h^-1 ^in LB medium at 37°C or 45°C. GRSW2-B1, as a marine bacterium, could grow well, with a similar growth rate in LB medium (0.5% w/v NaCl) and in LB medium containing high salt concentration up to 6%w/v NaCl, and thus can be classified as a moderate halotolerant bacterium (Margesin and Schinner 2001).

GRSW2-B1 was then challenged for its solvent tolerance by exposing high-density, late-exponential-grown cells to various types of solvent, including butanol, at high concentration (5%v/v), according to the technique previously reported ([Bibr B23][Bibr B25]). In addition, it is necessary to distinguish the solvent tolerance characteristic of *B. subtilis *GRSW2-B1 from that of a model Gram-positive bacterium and a type strain, *Bacillus subtilis *168 (Harwood and Wipat 1996); therefore the test of both strains was conducted in parallel. In comparison to *B. subtilis *168, GRSW2-B1 clearly exhibited higher tolerance to a broader range of solvents, with remarkable tolerance to alcohol groups in particular (Table 3).

**Table 3:** Organic solvent tolerance of *B. subtilis *GRSW2-B1 and *B. subtilis *168

		**Cell viability*^a^***			
		
**Organic solvent*^b^***	**Log *P*_ow_*^c^***	***B. subtilis *168**	***B. subtilis *GRSW2-B1**	***B. subtilis *GRSW2-B1** **/pHZT-PX**	***B. subtilis *GRSW2-B1** **/pHZK-PX**
					
None (control)	-	+++++++++	+++++++++	+++++++++	+++++++++
Octane	5.18	++++	+++++	++++	++++
Heptane	4.66	++++	+++++	++++	+++
Decanol	4.23	±	++++	++	++++
Hexane	3.90	+++	+++	++	+++
Nonanol	3.77	±	++++	±	+++
Cyclohexane	3.44	++	+++	++	+++
*m*-Xylene	3.20	++	++++	±	+++
*p*-Xylene	3.15	++	++++	±	+++
*o*-Xylene	3.12	++	++++	±	+++
Octanol	3.00	++	++++	±	+++
Toluene	2.73	++	++++	±	++++
Heptanol	2.62	±	++++	±	++++
Benzene	2.13	++	++++	±	+++
Hexanol	2.03	++	++++	±	++++
Butyl acetate	1.78	++	+++	±	+++
Pentanol	1.51	±	++++	±	++++
**Butanol**	**0.88**	**±**	**++++**	**±**	**++++**
Ethyl acetate	0.73	+++	++++	±	+++
THF (160 mM)	0.46	+++++++++	+++++++++	+++++++++	++++++++
Propanol	0.25	++++++	+++++	+++++++	+++++
2-Propanol	0.05	++++++++	++++++++	++++++++	++++++++
Ethanol	-0.31	+++++++++	+++++++++	+++++++++	++++++++
Acetonitrile	-0.34	++++++++	++++++++	++++++++	+++++++++
Methanol	-0.77	++++++++	+++++++++	++++++++	+++++++++
DMSO	-1.35	+++++++++	+++++++++	++++++++	++++++++

^a^Cells were initially grown to late-exponential phase in LB medium before organic solvent (5% v) was added. Cell viability was examined after 6 h of solvent exposure. The number of viable cells is represented by symbols +. The number of plus sign is corresponded to cell numbers (CFU.ml^-1^): ± ( < 1 × 10^2^); ++ (1 - 9 × 10^2^); +++ (1 - 9 × 10^3^); ++++ (1 - 9 × 10^4^); +++++ (1 - 9 × 10^5^); ++++++ (1 - 9 × 10^6^); +++++++ (1 - 9 × 10^7^); ++++++++ (1 - 9 × 10^8^); +++++++++ (1 - 9 × 10^9^). Data are means of the results from at least three individual experiments.

*^b ^*THF, tetrahydrofuran; DMSO, dimethylsulfoxide.

*^c ^*Log *P*_ow _value was obtained from KOW WIN version 1.67, EPI suite (U.S. Environmental Protection Agency).

**Figure 1:**
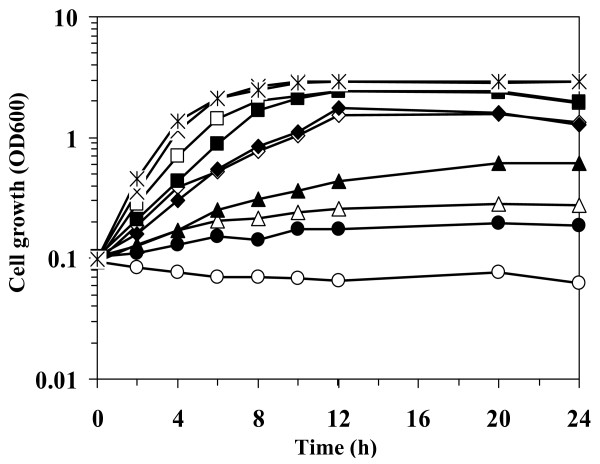
**Growth of *B. subtilis *GRSW2-B1 when butanol was added simultaneously with bacterial inoculum in LB medium**. Growth of non-acclimatized cells (opened symbol) and acclimatized cells (closed symbol) (expressed as logarithm scale of optical density at 600 nm) was monitored in the absence (×,✶) or presence of various concentrations of butanol (%v): 1.5 (□,■), 1.75 (◇, ◆), 2 (Δ▲), and 2.25 (○,●). Data are means of the results from at least three individual experiments.

Generally, the test procedure for solvent tolerance characteristics of bacteria is determined by exposing a solvent to high-density late-exponentially grown cells, as described earlier. However, in the fermentation process, it is also crucial to examine cell ability to tolerate and grow from its initial vulnerable stage of growth in the presence of a toxic substrate or product. Therefore, in this case, growth of GRSW2-B1 was dynamically monitored when butanol was added simultaneously with the bacterial inoculum (Figure 1). Despite the result showing that butanol has a negative effect on cells under growing conditions, GRSW2-B1 was able to cope with butanol toxicity and grow in the presence of butanol up to 2.0%v/v (Figure 1, opened symbol).

### Improvement of butanol tolerance of GRSW2-B1

Solvent tolerance of the host can be improved by two approaches: modification of medium composition and cell adaptation. It has been described that bacterial solvent tolerance could be enhanced by supplementation of amino acids, sugar and/or cell-energy-providing nutrients because they increase cell energy supply and thus increase efflux-pump-dependent solvent tolerance (Rűhl et al. 2009; Segura et al. 2005). Moreover, addition of salt has been proven to induce activity of efflux pump protein in halophilic and halotolerant bacteria (Tokunaga et al. 2004). Therefore, enhancement of solvent tolerance of GRSW2-B1 was attempted by cultivating cells in LB medium supplemented with artificial seawater nutrients (including vitamins and amino acids) and 2.75%w/v NaCl (Segura et al. 2008). Nevertheless, no significant improvement in solvent tolerance in GRSW2-B1 was observed using this modified medium.

Another approach to enhance solvent tolerance is cell acclimatization, in which cells are adapted to a toxic substance under particular conditions. In this study, GRSW2-B1 was repetitively acclimatized with butanol for 30 cycles (hereafter referred to as acclimatized cells). The butanol-acclimatized cells exhibited growth rates and final cell biomass similar to that of non-acclimatized cells in LB medium (Figure 1); whereas their butanol tolerance was substantially enhanced, as shown by their capability of growing in the presence of up to 2.25%v/v butanol (Figure 1, closed symbol). In each test, the viability of cells was also confirmed by colony counting. The optical density (OD_600_) of cells grown in the presence of 2.25%v/v butanol at 10 h of growth was approximately 0.2, which corresponded to viable cells with 7 ± 1 × 10^5 ^CFU·ml^-1^. No spore formation was observed up to 10 h of growth under the conditions tested. Our current results thus reveal that GRSW2-B1 has superior tolerance to butanol, when cells were either at late-exponential growth phase or grown from the initial stage of growth.

### Development of genetic transformation of butanol-tolerant GRSW2-B1

In addition to butanol tolerance, genetic tractability of the selected bacterium is an essential trait for the development of an alternative host for butanol production. Although there are diverse methodologies for transformation and gene expression in Gram-positive bacteria, it is known that many *Bacillus *sp. are extremely difficult to transform, and some of the recalcitrant strains remain untransformable despite testing with several currently available techniques. In spite of the difficulties, the development of an effective genetic transformation protocol is important for engineering a bacterial host for bioproduction, especially to a potential host with unique physiological properties, such as butanol-tolerant bacteria.

Accordingly, several cell pretreatment and transformation procedures were exhaustively conducted and adjusted for each butanol-tolerant bacterium previously isolated (i.e. GRSW1-B1, GRSW2-B1, CPSW1-B1 and CPSW2-B1). However, because of the natural recalcitrance of individual *Bacillus *sp., and probably the unique membrane characteristics of OSTB, attempts to transform GRSW1-B1, CPSW1-B1 and CPSW2-B1 have not yet been successful. On the other hand, electroporation was successfully applied for GRSW2-B1 transformation. Therefore, a number of parameters were optimized to prepare GRSW2-B1 electro-competent cells (i.e. growth phase, cell density, and electroporation buffer) and to achieve high efficiency of pHY300PLK plasmid DNA uptake by electro-transformation (i.e. electroporation conditions, plasmid DNA concentration, recovery medium and recovery period). Composition of the electroporation buffer is one of the most critical factors affecting electro-transformation efficiency. In this case, it exhibited a significant influence on cell competency and transformation efficiency of GRSW2-B1. The presence of sucrose and Mg^2+ ^in HSMG buffer increased the transformation efficiency by 20%, 50% and 70% over those in HS buffer, glycerol solution, and water, respectively. Mg^2+ ^and sucrose typically promote electro-transformation efficiency and cell viability because they stabilize the cell membrane from temporary distortion due to a high-voltage electric field, although they are not ascertainably advantageous for all bacteria (Wang and Griffiths 2009). The highest transformation efficiency of butanol-tolerant GRSW2-B1 at 5.17 × 10^3 ^CFU (μg DNA)^-1 ^was achieved when the competent cells were prepared from cells grown in LB medium to late-exponential phase with OD_600 _of 0.6, and washed with ice-cold HSMG buffer. Plasmid DNA of pHY300PLK was then introduced at 200 ng to the competent cells, and chilled on ice for 20 min before electroporation was performed at 25 μF, 200 Ω, with the optimized field strength at 10.5 kV·cm^-1^, yielding a time constant of 4.7 ± 0.1 ms. Then, an osmotically well-balanced TSB-plus medium was immediately added to the pulsed cells and incubated for 3 h - to reseal the membrane permeability and for recovery of the transformants - before spreading on LB medium agar plates including an appropriate antibiotic (i.e. tetracycline at 10 μg·ml^-1 ^or kanamycin at 5 μg·ml^-1^).

### Promoter strength of the expression vector in butanol-tolerant GRSW2-B1

**Figure 2:**
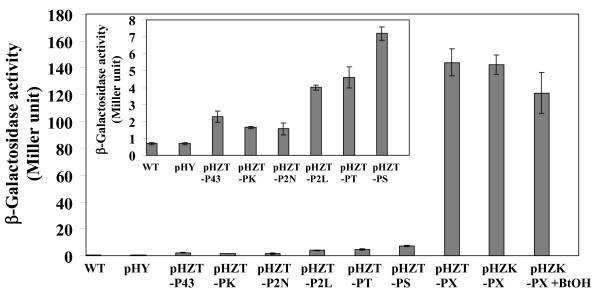
**Promoter-driven β-galactosidase activity**. *B. subtilis *GRSW2-B1, harboring each constructed expression vectors, was grown in LB medium to the same OD_600 _of approximately 0.8, and induced with the optimal induction condition of each promoter (if necessary) (as described in text). pHZT and pHZTK is pHY300PLK, carrying *trpA*, MCS, *lacZ*, with Tc^r ^and Km^r^, respectively. P43, PK, P2N, P2L, PT, PS, PX are P_43_, P_kan_, P_2N_, P_2L_, P_TetL_, P_Sapc_, and P_xylA _promoters (as described in details in Table 2). BtOH is butanol, which was added at 1% v/v. Inset is the enlarged *y*-axis scale to elaborate differences of the first eight data values. Data are means of the results from at least three individual experiments.

The achievement of bioproduction of industrial chemical and biofuel, e.g. butanol, in a heterologous host also relies on a promoter-mediated gene expression system. A suitable promoter for efficient production of recombinant gene products is considered based on its strength and controllability (i.e. inducibility) at an indicated time or condition (Timmis et al. 1994). In this study, the following prominent promoters of *Bacillus *sp. and Gram-positive bacteria, which could be classified into two groups, were introduced into pHY300PLK, an *E. coli*-*Bacillus *shuttle vector, and their activity was then assessed by measuring β-galactosidase reporter gene activity. The first group of promoters consisted of constitutive promoters including: P_43_, a well-characterized promoter that is functional during both exponential and stationary growth phases (Wang and Doi 1984); P_Km_, a promoter of the kanamycin resistance gene (Masai et al. 1995); and P_2N_, a strong promoter that functions in *Brevibacillus choshinensis*. The second group comprised inducible promoters, consisting of: P_2L_, a temperature-inducible promoter (Li et al. 2007); P_TetL_, a strong promoter of the *tetL *gene encoding efflux-mediated tetracycline resistance in *Streptococcus*, *Enterococcus*, and *Bacillus *(Butaye et al. 2003); P_spac_, an IPTG-inducible promoter (Vagner et al. 1998); and P_xylA_, a xylose-inducible promoter originated from *B. megaterium*. The activity of constitutive promoters (P_43_, P_Km _and P_2N _in pHZT-P43, pHZT-PK and pHZT-P2N, respectively) in GRSW2-B1 was slightly higher (two- to threefold) than the basal activity of the wildtype and the wildtype harboring an original vector (i.e. pHY300PLK) (Figure 2). The expression activity of an inducible promoter in GRSW2-B1 was tested at each optimal inducible condition. P_2L _is a temperature-inducible promoter, whose activity at 45°C was 2.3-fold higher than that at 37°C. P_TetL _is a strong constitutive promoter of the *tetL *gene commonly found in Gram-positive bacteria. The induction of this promoter is possible, but is not strictly required, because it does not involve a binding of tetracycline to a repressor protein as is generally reported in P_TetA_, a well-characterized, widely distributed promoter among Gram-negative bacteria (Butaye et al. 2003). Nonetheless, in this study the addition of tetracycline, mainly to stabilize the vector, may positively influence the induction of this promoter as well. P_spac _(in pHZT-PS) exhibited the maximum inducible activity when 2 mM IPTG was included. The activity level of these promoters (P_2L_, P_TetL _and P_spac_) was approximately six- to tenfold of the basal activity (Figure 2, inset). On the contrary, a significant level of β-galactosidase activity was observed in the transformants harboring pHZT-PX, where the activity was 206-fold higher than that of the basal activity (Figure 2). The addition of xylose at 0.1% w/v as an inducer enhanced the activity by 1.5-fold, whereas the addition of glucose, with the concentration ranging from 1-40 g L^-1^, had no effect on the activity (data not shown).

### Effect of efflux-mediated tetracycline resistance determinant, TetL, on solvent tolerance of GRSW2-B1

Prior to the construction of an expression vector suitable for GRSW2-B1, its antibiotic resistance was initially tested to select the antibiotic resistance genetic marker. GRSW2-B1 is not resistant to tetracycline; therefore a commercially available pHY300PLK, harboring the tetracycline resistance gene (*tetL*), was chosen (Table 3). Because pHZT-PX yielded the highest level of gene expression, it was initially selected as a potential expression system to advance its genetic modification. Nonetheless, prior to any further genetic engineering, solvent tolerance of the transformants was reaffirmed. Unexpectedly, tolerance of the transformants/pHZT-PX to solvents, with log *P_ow _*value ranging from 0.73-4.23, was drastically reduced (Table 3). Previous reports have shown that an antibiotic resistance system may have cross-activity with bacterial tolerance to structurally unrelated toxic chemicals including solvents (Fernandes et al. 2003); therefore, contrary to the obtained results, enhancement of solvent tolerance in GRSW2-B1/pHZT-PX as a result of the introduction of *tetL*, forming TetL, was initially anticipated. In order to inspect whether the reduction of solvent tolerance was caused by TetL, the *tetL *gene in pHZT-PX was replaced by the kanamycin resistance gene (*kan*), forming pHZK-PX. The replacement resulted in full recovery of solvent tolerance of GRSW2-B1 (Table 3) and did not adversely affect gene expression level (Figure 2). This result showed that the presence of TetL certainly conferred tetracycline resistance to GRSW2-B1, but it caused substantial reduction of solvent tolerance.

Further investigation was conducted to determine if the gene expression of pHZK-PX could be maintained in the presence of butanol stress. In the presence of 1%v/v butanol, gene expression level was maintained at a level comparable to that in the absence of butanol (Figure 2). This result demonstrates the potential application of this expression system in butanol production using butanol-tolerant GRSW2-B1 as an engineered host.

### Segregational stability of the expression vector in butanol-tolerant GRSW2-B1

Another important aspect of large-scale fermentation using an engineered microbial host is the prevention of contamination. As previously stated, the use of an antibiotic in such fermentation may be necessary, but it is generally undesirable due to economic reasons and the problem of microbial antibiotic resistance (Fischer et al. 2008). Therefore, segregational stability of the expression vector pHZK-PX in butanol-tolerant GRSW2-B1 transformants was evaluated in the presence and absence of butanol stress. The result showed that, in the presence and absence of butanol, 95 ± 0.7% and 91 ± 0.8% of the constructed expression vector pHZK-PX could be stably maintained in GRSW2-B1, respectively.

## Discussion

The aim of this work was to search for and develop a butanol-tolerant bacterium as a genetic-recombinant host for further application in bioproduction of alcohol-biofuel, initially focusing on butanol. Because butanol is classified as an extremely toxic chemical to microorganisms, its toxicity becomes the primary problem for its production via microbial fermentation. Numerous studies have been conducted to find, modify and construct an optimal host with high tolerance to butanol. While the construction of a butanol biosynthesis pathway in several heterologous hosts has been reported, the major obstacle limiting their achievement is due to low tolerance of the host to butanol toxicity (Fischer et al. 2008).

In this study, GRSW2-B1 was isolated as butanol-tolerant bacterium. It exhibited a distinct tolerance to butanol at higher concentration when compared to that of *B. subtilis *168, a type strain which has been extensively used as an industrial heterologous host. Moreover, GRSW2-B1 also showed higher butanol tolerance than *B. subtilis *KS438, which could tolerate butanol up to 1.25%v/v and was earlier engineered for butanol production (Nielsen et al. 2009). This result illustrated that butanol tolerance is a strain-specific property (Sardessai and Bhosle 2002).

To assess the solvent tolerance of bacteria, there are three reported approaches. The first one involves overlaying a solvent onto a medium agar plate or slant which was previously inoculated with bacteria colonies (Li et al. 1998). This technique is less sensitive and has generally been used for primary screening of OSTB. The other approaches involve a solvent tolerance test in liquid medium. The most extensively used technique to characterize bacterial solvent-tolerance is by exposing a high-density suspension of cells, previously grown to late-exponential phase, to a solvent for a certain period of time, and then determining viable cell numbers. According to this test result, GRSW2-B1 showed remarkable tolerance ability to butanol (up to 5%v/v), which is an attractive characteristic for a potential host.

Nevertheless, in the fermentation process where a toxic substrate is initially presented or a toxic product is gradually formed, it is crucial to examine cell ability to tolerate and grow from its vulnerable stage of growth in the presence of the toxic substrate or product. This technique is to assess the solvent tolerance of bacteria during the so-called growing (or culturing) condition. In this test, GRSW2-B1 was able to grow from 1%v/v of cell inoculum, and overcome the toxicity of butanol, presented at 2%v/v. This result clearly shows a distinct tolerance characteristic of GRSW2-B1 because this butanol level is significantly higher than the level that other *Bacillus *sp. could defeat, when tested under growing-conditions. For instance, *Bacillus *sp. SB1 isolated from mangrove sediment was reported to have a 92% reduction in growth rate when grown in the presence of 2%v/v butanol (Sardessai and Bhosle 2002).

Our current results reveal that GRSW2-B1 has superior tolerance to butanol when cells are either at late-exponential growth phase or grown from the initial stage of growth. This characteristic is advantageous for a potential genetic vehicle, where specific biosynthesis genes of the target product can be endowed in a suitable expression vector, in which a variety of regulatory controls may be employed. Moreover, this prominent tolerance opens up more opportunities for a recombinant host to be applied in an appropriate fermentation process, using either growing cells or high-density resting cells, with different types of expression and process controls, e.g. batch, fed-batch, continuous or multi-stage continuous (Garcia et al. 2011).

Nevertheless, prior to achieving the goal of host development, the prerequisite properties of a potential bacterium, i.e. genetic manipulation and gene expression efficiency, were characterized and optimized. Although several genetic transformation protocols of *Bacillus *sp. have been reported, they tend to be host-specific and depend upon empirical observations, and their success relies on a variety of factors (Fischer et al. 2008). Despite the difficulties, genetic transformation of GRSW2-B1 was proven feasible and was optimally established in this study. In addition, because a promoter plays a central role as a regulatory element of expression of the desired genes for bioproduction, it is important to seek the best match between host and the promoter. Our results reveal that a xylose promoter yields the highest level of gene expression. This result is in agreement with previous reports, in which a xylose promoter frequently yields high-level heterologous gene expression in *B. megaterium *and *B. subtilis *(Terpe 2006). Nevertheless, an effective and suitable expression system is not only judged by the promoter - whether it can be recognized by the host and how well it can drive gene expression to a reasonable level - but it is also often a consideration of the type of target protein. The strongest promoter driving a high level of expression may not always be the most suitable, because some gene products may be toxic to host cells, even when synthesized at low levels. In this study, we demonstrated that the gene expression in butanol-tolerant GRSW2-B1 could be effectively driven by several promoters with different levels of gene expression. The highest expression was observed with P_xylA _promoter.

While the constructed expression vector pHZT-PX yielded the highest expression level, the GRSW2-B1 host harboring this vector suffered severely from the reduction of butanol tolerance, caused by efflux-mediated tetracycline determinant, TetL (*tetL *gene product). TetL is one of the tetracycline resistance determinants, distributed mainly in Gram-positive bacteria. Its resistance mechanism involves an energy-dependent efflux transporter system where energy-dependent membrane-associated proteins export tetracycline as well as toxic chemicals out of cells (Roberts 1996). Therefore, the adverse effect of TetL on solvent tolerance in GRSW2-B1 was strikingly unpredicted. This result may suggest that TetL is not originally involved in solvent tolerance in this bacterial strain or, if it is present, the increase of TetL protein dosage (through the expression of the *tetL *gene in the expression vector) may interfere with the solvent tolerance mechanism and thus cause severely adverse effects on its tolerance. Alternatively, studies have revealed that TetL is a multifunctional protein which is also responsible for the efflux of a divalent-cation-tetracycline complex in coupled-exchange fashion for protons (i.e. metal-tetracycline/H^+ ^antiporter) and also enhances Na^+^/H^+ ^antiporter activity in *B. subtilis *(Guffanti and Krulwich 1995). Since previous studies indicated the role of divalent cations (i.e. Ca^2+ ^and Mg^2+^) in stabilizing the cell membrane and reducing the charge repulsion between anionic molecules in the cell membrane, which significantly facilitates bacterial solvent tolerance (Aono et al. 1994;Inoue et al. 1991), the introduction of TetL may cause alteration of the divalent-cation concentration surrounding cells, which interferes with cell membrane stabilization and leads to the drastic reduction of solvent tolerance of GRSW2-B1/pHZT-PX. Although the influence of TetL on solvent tolerance of GRSW2-B1 remains to be further investigated, this study is the first to describe the adverse effect of the efflux-mediated antibiotic resistance determinant, TetL, on the solvent tolerance of bacteria.

In conclusion, since the role of higher alcohols (e.g. butanol) as advanced biofuels has become increasingly important, this has led to high demands for alternative microbial hosts with solvent-tolerant-traits. GRSW2-B1 is reported as a newly isolated, butanol-tolerant bacterium. It is capable of tolerating butanol as well as a range of solvents, especially alcohol groups. Not only does it has distinct solvent tolerance and genetic modification susceptibility characteristics, but *B. subtilis *also shares phylogenetic similarity with *Clostridium*, a native strain for butanol production. Therefore, *B. subtilis *GRSW2-B1 is markedly attractive to be further engineered and established as a genetic host for bioproduction of butanol.

## Lists of abbreviations

HS buffer: (1 mM HEPES buffer containing 250 mM sucrose, pH 7.0); HSMG buffer: (HS buffer with 1 mM MgCl_2 _and 10% glycerol, pH 7.0); GRSW2-B1: (*Bacillus subtilis *strain GRSW2-B1); OSTB: (organic-solvent tolerant bacteria);

## Competing interests

The authors declare that they have no competing interests.

## Authors' contributions

NK participated in the design of the study, performed the experimental work and data interpretation. WR participated in bacterial screening. TT, JK and ASV participated in the design of the study and analysis of the data. ASV wrote the manuscript and all authors participated in commenting and revising it. All authors contributed to the scientific discussion throughout the work and have read and approved the final manuscript.
